# Screening of Organic Acid Type and Dosage in Drinking Water for Young Rabbits

**DOI:** 10.3390/ani14152177

**Published:** 2024-07-26

**Authors:** Adrián Ramón-Moragues, Chiara María Vaggi, Jorge Franch-Dasí, Eugenio Martínez-Paredes, Catarina Peixoto-Gonçalves, Luis Ródenas, Maria del Carmen López-Luján, Pablo Jesús Marín-García, Enrique Blas, Juan José Pascual, María Cambra-López

**Affiliations:** 1 Institute of Animal Science and Technology, Universitat Politècnica de València, Camino de Vera s/n, 46022 Valencia, Spain; jorfrada@upv.es (J.F.-D.); macamlo@upvnet.upv.es (M.C.-L.); 2 ERP Unit of Nutritional Physiology, University of Veterinary Medicine, 1210 Vienna, Austria; chiaramariavaggi@gmail.com; 3 Department of Animal Production and Health, Veterinary Public Health and Food Science and Technology, Veterinary School, Universidad Cardenal Herrera-CEU, 46115 Valencia, Spain

**Keywords:** growing rabbits, additives, organic acids, gastric pepsin, digestive health

## Abstract

**Simple Summary:**

This study aimed to evaluate rabbits’ responses to the administration of six organic acids (OAs) through drinking water at three concentrations (pH levels). Animal drinking and feeding behaviour, pH evolution along the animal’s gastrointestinal tract, and gastric pepsin activity were monitored in post-weaning rabbits for seven days. The screened combinations were used to identify the optimal OA candidates and pH values, while eliminating those that may exhibit early adverse effects in young rabbits. During our short-term assessment period, the OAs with the greatest potential for use in rabbit farming were formic acid, acetic acid, and citric acid at pH 4. However, further validation with a larger population of animals, and extending the duration of OA supplementation during the growing period could improve performance outcomes and enhance the modulation effect of the gastrointestinal environment.

**Abstract:**

Organic acids (OAs) are employed in animal feed to regulate gastrointestinal disorders and diarrhoea thanks to their ability to modulate the gastrointestinal environment and their antimicrobial capacity. However, there is not enough evidence regarding the most adequate OA and its effectiveness in rabbit farming. Therefore, the aim of this study was to screen and evaluate the response of young rabbits to six OAs, administered via drinking water, at three different concentrations (pH levels). Organic acids (acetic, ACET; formic, FOR; propionic, PROP; lactic, LAC; citric, CIT; and butyric, BUT) were tested at three concentrations (pH 3, 4, and 5). A negative control (CON; non-acidified water) was also included. We used 240 weaned rabbits (28 days old) divided into 2 batches. In each batch, animals were randomly allocated to 1 of the 19 experimental treatments and were housed in group cages of 6 animals per cage, treatment, and batch. Among the 240 rabbits, an additional cage with 6 animals was included to determine the initial physiological state of the animals. All animals were fed with commercial pelleted feed throughout the whole experiment. The duration of the study was 7 days, until 35 days of age. At 31 and 35 days of age, in each batch, three animals per day and treatment were slaughtered. The pH of the digestive contents in the fundus, antrum, duodenum, jejunum, ileum, and cecum, as well as the gastric pepsin enzyme activity, was measured. Water and feed consumption per cage and individual body weight (BW) were recorded daily. The type and dosage of OAs affected water intake. ACET 3, PROP 3, and BUT 3 reduced water intake compared to CON, negatively impacting feed intake and weight gain. FOR and CIT acids led to the highest BW and weight gain at 35 days, compared to PROP, LAC, and BUT (*p* < 0.05); showing ACET intermediate values. While OAs had limited effects on gastric and small intestine pH, acidified water at pH 4 and 5 lowered ileum and caecum pH (*p* < 0.05) compared to pH 3. Acidified water at pH 4 showed the highest (*p* < 0.05) pepsin activity compared to pH 3 and pH 5. Considering the limited sample size and short-term assessment period of our screening test, the OAs with the highest potential for use in post-weaning rabbits were FOR, ACET, and CIT at pH 4. The selected combinations did not exhibit any early adverse effects in young rabbits. These results should be further confirmed in a broader population of animals. It would also be advisable to extend the application of OAs over longer periods to evaluate their effects throughout the entire growing period of rabbits.

## 1. Introduction

Weaning is a critical period for young rabbits. Ensuring their health and welfare during this time is key to improving post-weaning survival and performance and can impact overall farm profitability. Rabbit production is often affected by digestive disorders which frequently start within the first two weeks post-weaning [1]. These conditions result in pain and discomfort for the animals, increasing mortality, impairing animal welfare, and causing economic losses for producers [2].

Although the precise aetiology of digestive disorders, and more specifically epizootic rabbit enteropathy (ERE), remains uncertain, some studies suggest that a bacterial origin is likely [1,3,4]. The post-weaning period is associated with changes in intestinal commensal microbial communities and microbiota instability [5]. These conditions may facilitate the development of ERE pathogenesis. Although no single bacteria has been identified as the aetiological agent of ERE, *Escherichia coli* and *Clostridium perfringens* are very frequently isolated in high numbers in rabbits suffering ERE [3]. 

In the past, one of the most common methods for controlling and treating ERE was the use of antibiotics. However, administering these drugs to livestock at low doses and for extended periods of time aggravated the emergence of bacterial resistance [6]. This situation has prompted concerns about the loss of effectiveness of antibiotics in the treatment of infections in both livestock and human health [7]. In response, the European Union—through Regulation (EU) 2019/6 on veterinary medicinal products [8]—restricted the use of these drugs to reduce the spread of antimicrobial resistance in animals. Although for several years rabbit farms have been adopting a combination of feeding strategies (such as feed restriction and the use of low-starch–high fibre diets), and management practices (such as improved hygiene practices, biosecurity measures, and environmental control) to combat ERE; there is still a need for integrated measures that can successfully prevent and control ERE and other digestive disorders other than antibiotics. 

In general, enteropathogens enter the body orally. Therefore, the initial protective barrier that pathogens must overcome is the animal’s stomach [9]. The strong acidic environment of the stomach inhibits the growth of microorganisms [10]. However, the capacity of young rabbits to acidify their stomachs after weaning is limited. Hydrochloric acid secretion typically begins to increase at 16 days of age, reaching a stable level around 30 days of age [11]. In addition, the high buffering capacity of rabbit feed exacerbates this condition and impacts gastric pH [11,12]. The increase in intraluminal stomach pH due to the lack of hydrochloric acid synthesis results in the loss of the protective barrier function against the entry of pathogens [9], allowing their passage to the distal digestive tract and favouring their subsequent colonization [3,13,14]. This situation, in conjunction with an insufficient digestive enzyme activity due to the immaturity of the digestive system [15,16], can result in poor nutrient digestion after weaning, thereby increasing the risk of enteropathy [17].

Consequently, there is a need for preventive measures that can contribute to enhancing the gastric barrier in rabbits and reducing the incidence of post-weaning digestive disorders. One potential solution to compensate for the deficit of gastric acidification and reinforce this barrier against pathogens is the use of organic acids (OA) [18]. These compounds can lower the pH when they release their hydrogen ions. Moreover, they have been shown to inhibit bacterial growth [19]. Undissociated OAs are capable of passing through the bacterial cell wall and membrane and tend to dissociate in the bacterial cytoplasm due to its neutral pH [18]. As more acid molecules are dissociated, the cytoplasmic pH will become low. Free protons can alter microbial metabolism by inhibiting enzymes and transport systems, resulting in bacterial lysis, and the inhibition of bacterial growth [19]. 

As outlined by Partanen and Mroz [9], the most promising OAs as inhibitors of microbial growth used in livestock farming are formic, acetic, propionic, butyric, lactic, sorbic, fumaric, tartaric, and citric acids. These OAs are characterized by a pKa ranging from 3 to 5, meaning they are weak OAs. The pKa is a constant that expresses the pH at which half of the acid molecules are dissociated. The higher the pKa value, the weaker the acid. Therefore, the objective of OAs is to reduce gastric pH and to inhibit bacterial growth. However, the antimicrobial and acidifying capacity/potential of OA in animals varies depending on the type of acid, molecular weight, pKa and its concentration, as well as the administration route, and the buffering capacity of the diet when administered via feed. 

The incorporation of OAs into animal feed has been extensively tested in pigs and poultry, and to a lower extent in other species including rabbits. Their use in pigs has been shown to enhance animal’s productive efficiency and to reduce diarrhoea incidence during post-weaning and growing periods [9,20,21]. Similarly, in poultry, the use of these compounds has resulted in favourable outcomes, including improvements in feed conversion rates and digestive enzyme efficiency [22,23]. However, there is scarce literature on the use of OA in growing rabbits. Due to rabbit’s peculiar digestive physiology, it is not possible to extrapolate results from pigs and poultry. Furthermore, the data available on rabbits are contradictory [19]. Moreover, some OAs are known to possess peculiar organoleptic characteristics, including a strong odour and taste (e.g., acetic and butyric acid). These characteristics may influence animals’ feeding and drinking behaviour. However, the effect of these compounds on feeding behaviour in rabbits has not yet been described. Consequently, further research is required to determine the effects of OAs when used in growing rabbits, particularly in terms of performance and digestive health, to select the most adequate OA for this species and eliminate those that are not suitable. 

Thus, our study aimed to screen and evaluate the response of young rabbits to six OAs, administered via drinking water, at three different concentrations (pH levels). Animal drinking and feeding behaviour, the pH evolution along the animal’s gastrointestinal tract, and gastric pepsin activity were monitored in post-weaning rabbits during seven days. The screened combinations were used to identify the optimal OA candidates and pH values, while eliminating those that may exhibit early adverse effects in young rabbits.

## 2. Materials and Methods

### 2.1. Ethical Considerations

The experiment was conducted at the experimental growing rabbit farm of the Universitat Politècnica de València (UPV, Valencia, Spain). The experimental procedure was reviewed and approved by the Animal Welfare Ethics Committee of the UPV (code number 2022 VSC PEA 0111). Additionally, the trial followed the specifications of the Directive 2010/63/EU of the European Parliament and of the Council of 22 September 2010 on the protection of animals used for scientific purposes.

### 2.2. Experimental Treatments

A total of six OAs (acetic, formic, propionic, lactic, citric, and butyric) administered through drinking water were tested (Table 1). For each acid, three different dosages were employed to achieve pH 3, 4, and 5 in the drinking water. Additionally, a control group (CON) with tap water was included. This resulted in a total of 19 experimental treatments in drinking water: acetic acid (ACET 3, ACET 4, ACET 5), formic acid (FOR 3, FOR 4, FOR 5), propionic acid (PROP 3, PROP 4, PROP 5), lactic acid (LAC 3, LAC 4, LAC 5), citric acid (CIT 3, CIT 4, CIT 5), butyric acid (BUT 3, BUT 4, BUT 5), and CON.

### 2.3. Animals and Housing Conditions 

A total of 240 rabbits of both sexes of the LP genetic line of the UPV [24], from different litters, were used. Animals were weaned at 28 days of age (average body weight (BW) = 533.8 ± 16.5 g). The experiment was conducted in two batches (120 rabbits/batch). The duration of the experiment was seven days per batch, from 28 to 35 days of age. 

**Figure 1:**
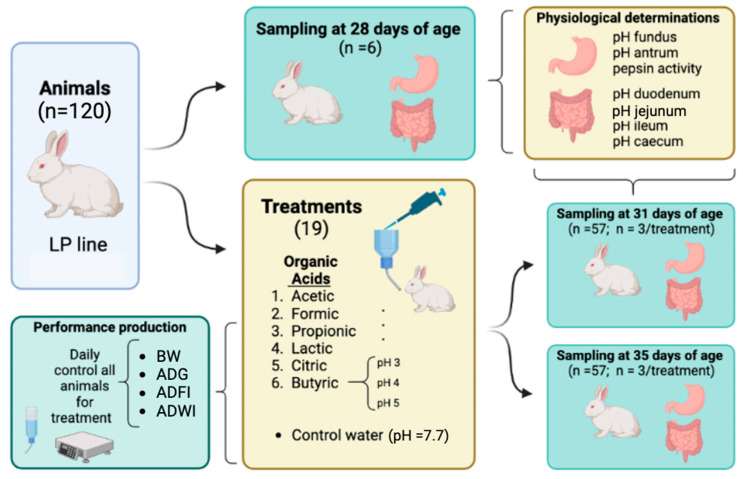
Schematic overview of experimental design in each batch (created with Biorender.com: https://app.biorender.com/; accessed on 10 May 2024).

At the start of the experiment, after weaning, animals were randomly distributed into 19 group cages (L: 50 × W: 80 × H: 32 all in cm) with six animals. Each cage was assigned to an experimental treatment, with one replicate per treatment and batch (total of 2 cages and 12 animals/treatment). Additionally, an extra cage with six animals was utilized in each batch to ascertain the initial (baseline) physiological status of animals at 28 days of age. A schematic overview of the experimental design is presented in Figure 1.

All animals were fed a commercial feed for growing rabbits manufactured with pellet size 2.5–3.5 mm × 5.0–8.0 mm based on alfalfa meal, wheat bran, and sunflower meal. The diet was formulated to meet the nutrient requirements for growing rabbits [25], except for neutral detergent fibre, which exceeded those recommendations. This is a common practice on commercial farms, involving moderate-to-high levels of fibre to support gastrointestinal health and digestive function. The analysed chemical composition of the feed on dry basis was as follows: crude protein 16.5%, crude fat 4.1%, starch 17.4%, neutral detergent fibre 45.7%, acid detergent fibre 22.2%, acid detergent lignin 3.77% and ash 8.07%. 

Rabbits had ad libitum access to pelleted feed in a feeding trough and water (using a 2-L bottle drinker per cage) during the whole experiment. The facilities were equipped with an automated environmental control system. Temperature and ventilation rate were appropriate for the age of the rabbits. Indoor temperature varied from 16 to 24 °C. The light programme was set to 12 h of light and 12 h of darkness throughout the study. 

### 2.4. Recorded Parameters

To assess the impact of the use of OA in post-weaning rabbits, both performance and digestive physiological measurements were conducted on the animals. Regarding performance data, individual BW was measured three times: at 28 days (*n* = 12 animals per treatment), 31 days (*n* = 12 animals per treatment), and 35 days of age (*n* = 6 animals per treatment). From these data and number of animals, individual average daily gain (ADG) was calculated for each period and globally (from 28 to 31 days of age, from 31 to 35 days of age, and from 28 to 35 days of age). Average daily feed intake (ADFI) and average daily water intake (ADWI) were measured daily at 9:00 a.m., per cage, by weighing the drinkers and feeders. This was done to determine feeding behaviour and immediate animal’s response, particularly on drinking behaviour, as well as the potential adaptation to acidified drinking water with each of the tested OA. The number of animals present per cage in each period was used to correct and calculate ADFI and ADWI (6 animals per cage and batch for the period 28 to 31 days, and 3 animals per cage and batch for the period 31 to 35 days). ADWI:ADFI ratio was calculated for each pH level and acid type. 

Furthermore, on days 31 and 35 of age, three animals per cage were slaughtered in each batch to conduct digestive physiological measurements (6 animals per treatment at 31 days, and 6 animals per treatment at 35 days). Animals were euthanised by intracardiac injection of sodium thiopental (75 mg/kg BW). Euthanasia was conducted from 20:00 h to minimize the influence of caecotrophy on the composition of the digestive content [26]. Subsequently, the gastrointestinal tract was excised and divided into its constituent parts. The pH was measured along the gastrointestinal tract in the stomach (antrum and fundus), small intestine (duodenum, jejunum, and ileum) and caecum sections. In all sections, pH measurements were conducted immediately using a portable pH meter (Vio XS, XS Instrument, Carpi, Italy) with two types of electrodes. A steel electrode (sensor 2-Pore steel, XS Instrument, Carpi, Italy) was employed to ascertain stomach pH directly within the stomach cavity. Additionally, a microelectrode (XS sensor micro, XS Instrument, Carpi, Italy) was employed to ascertain the pH of intestinal and caecal samples removed from each section and placed into 5 mL Eppendorf tubes. Gastric content samples were also collected in 50 mL tubes and stored at −80 °C for subsequent determination of pepsin enzyme activity [27].

### 2.5. Statistical Analyses

Data on the evolution of ADFI and ADWI were statistically analysed using the proc MIXED of SAS (North Carolina State University, Raleigh, NC, USA) [28]. Sample size was two cages per treatment. Corrections in ADWI and ADFI per cage were done according to the animals present in each period, as described in the recorded parameters Section 2.4. The model included the fixed effects of treatment (19 treatments), day (29, 30, 31, 32, 33, 34, and 35 days of age), their interaction, and batch (1 and 2). The ADWI:ADFI ratio was analysed using proc MIXED procedure as well, including the fixed effects of treatment, day, and batch. The random effect in these models was the cage.

Individual animal BW and ADG data in each period were statistically analysed using proc GLM of SAS (North Carolina State University, USA) [28]. Sample size per treatment was 12 animals from 28 to 31 days of age, and 6 animals from 31 to 35 days of age, and globally. The model included the treatment (19 treatments) as fixed effect and batch (1 and 2) as covariate. Additionally, to evaluate the effect of acid type and pH, the same procedure was used, including acid type (ACET, FOR, PROP, LAC, CIT, and BUT), pH level (pH 3, pH 4, and pH 5), and batch (1 and 2) as fixed effects. Sample size per acid was 36 animals from 28 to 31 days of age, and 18 animals from 31 to 35 days of age, and globally. Sample size per pH was 72 animals from 28 to 31 days of age, and 36 animals from 31 to 35 days of age, and globally.

The digestive physiological measurements were statistically analysed using the using the proc MIXED of SAS (North Carolina State University, USA) [28]. Sample size for the measured digestive parameters was 6 animals per treatment and day. The model included the fixed effects of treatment (19 treatments), day of slaughter (31 and 35 days of age), their interaction, and batch (1 and 2). Additionally, to evaluate the effect of acid type and pH, the same procedure was used, including acid type (ACET, FOR, PROP, LAC, CIT, and BUT), pH level (pH 3, pH 4, and pH 5), and batch (1 and 2) as fixed effects. In these analyses, sample size was 18 animals per acid day and 36 animals per pH and day. The random effect in these models was the cage.

All data were presented as least-squares means. Differences were considered significant at *p* < 0.05. 

## 3. Results

### 3.1. Effect on Water and Feeding Behaviour 

**Figure 2:**
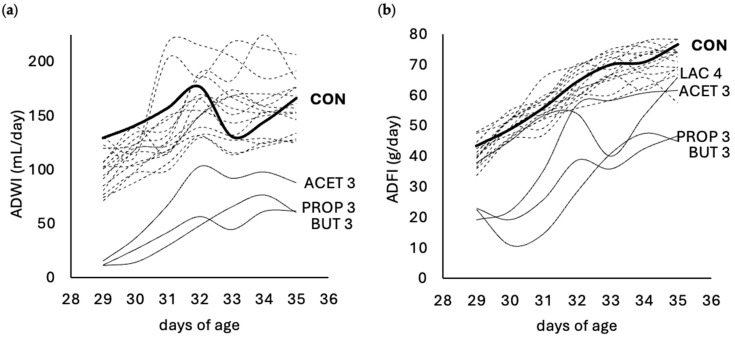
Evolution of average daily water intake (ADWI) (**a**) and average daily feed intake (ADFI) (**b**) per treatment throughout the trial, compared to the control group (continuous thick line). Dotted lines represent the remaining treatments, while the continuous thin line corresponds to treatments showing a statistically significant difference in intake compared to the control group. Experimental treatments ACID and pH level: acetic acid (ACET 3, ACET 4, ACET 5), formic acid (FOR 3, FOR 4, FOR 5), propionic acid (PROP 3, PROP 4, PROP 5), lactic acid (LAC 3, LAC 4, LAC 5), citric acid (CIT 3, CIT 4, CIT 5), butyric acid (BUT 3, BUT 4, BUT 5), and control (CON). *n* = 2 cages per treatment including 6 animals per cage (from 28 to 31 days of age) and 3 animals per cage (from 31 to 35 days of age).

Figure 2 illustrates the evolution of ADWI (a) and ADFI (b) throughout the experiment. During the experimental period, water intake progressively increased in all treatments. The ADWI of the animals in the CON group was 149 mL/day, with a minimum of 129 mL (day 29) and a maximum of 177 mL (day 32) (Figure 2a). Although our results come from two observations (cages), we could observe how the addition of some OAs initially altered water intake. The inclusion of ACET 3, PROP 3, and BUT 3 significantly reduced ADWI compared to CON (−76, −100, and −106 mL/day, respectively; *p* < 0.05). This resulted in a 2.1 to 3.4-fold decrease in ADWI. These differences with the CON group were particularly pronounced during the initial three days (from day 29 to 31). Animals in these treatments began to recover and increase their water intake from day 32 onwards, although they still exhibited water consumption levels that were significantly lower than those of the CON group. 

ADWI conditioned ADFI, both showing a similar trend. ADFI of the animals in the CON group was 61 g/day, with a minimum of 43 g (day 29) and a maximum of 77 g (day 35) (Figure 2b). Similarly to the findings for water, the addition of some OAs affected feed intake. The inclusion of ACET 3, PROP 3, and BUT 3 significantly reduced ADFI compared to CON (−16, −28 and −31 g/day, respectively; *p* < 0.05). Animals in the ACET 3 treatment demonstrated a faster recovery in feed intake, yet their consumption levels remained significantly lower than those of the CON group. Animals in the LAC 4 treatment showed a marked decrease in feed intake on days 33 to 34 of age compared to CON (−30 and −18 g/day, respectively; *p* < 0.05), increasing thereafter.

**Figure 3:**
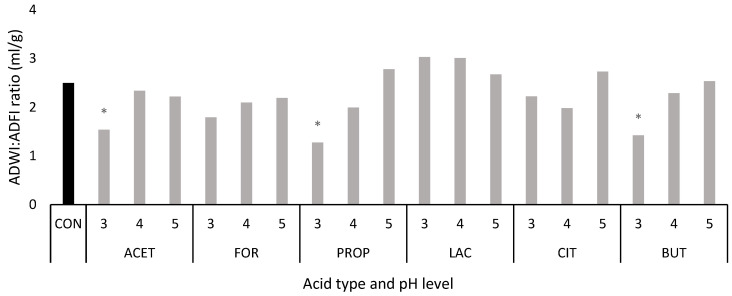
Calculated average daily water intake (ADWI): average daily feed intake (ADFI) ratio per treatment. * Indicates statistically significant differences with the control group. The experimental treatments comprised: acetic acid (ACET 3, ACET 4, ACET 5), formic acid (FOR 3, FOR 4, FOR 5), propionic acid (PROP 3, PROP 4, PROP 5), lactic acid (LAC 3, LAC 4, LAC 5), citric acid (CIT 3, CIT 4, CIT 5), butyric acid (BUT 3, BUT 4, BUT 5), and control (CON). *n* = 2 cages per treatment, including 6 animals per cage (from 28 to 31 days of age) and 3 animals per cage (from 31 to 35 days of age).

Figure 3 shows the ADWI:ADFI ratio in each treatment throughout the experiment compared to the CON group. The ratio ranged from 2.11 to 2.44 on average. The low ADWI—particularly in ACET 3, PROP 3, and BUT 3 groups—resulted in significantly lower ADWI:ADFI ratios compared to the CON group.

### 3.2. Effect on Growth Performance 

Table 2 shows the growth performance data for all treatments, compared to the CON group, over the seven-day experimental period. At the start of the experiment, animals were weaned at an average age of 28 days, with an average BW of 532 g. At 35 days of age, they reached 727 g regardless of the treatment they received. No animals died during the experiment. At 31 days of age, BW was significantly lower in ACET 3 (−61.2 g, *p* = 0.0345), PROP 3 (−94.1 g; *p* = 0.0012), and BUT 3 (−147.5 g, *p* < 0.0001) compared to the CON group. These differences in BW persisted at 35 days of age (−100 g ACET 3, −174.2 g PROP 3, and −228.4 g BUT 3 compared to CON; *p* < 0.05). Changes in BW were also reflected in ADG. In comparison to CON, ADG from 28 to 31 days of age was significantly lower in ACET 3, PROP 3, and BUT 3 (−20.9, −33.6, and −46.8 g/day, respectively; *p* < 0.0001). Results from the first days negatively impacted global ADG from 28 to 35 days of age in these treatments (−10, −24 and −26 g/day for ACET 3, PROP 3, and BUT 3 compared to CON, respectively, *p* < 0.05). There were no statistically significant differences in BW and ADG between the rest of experimental treatments and the CON group. 

**Figure 4:**
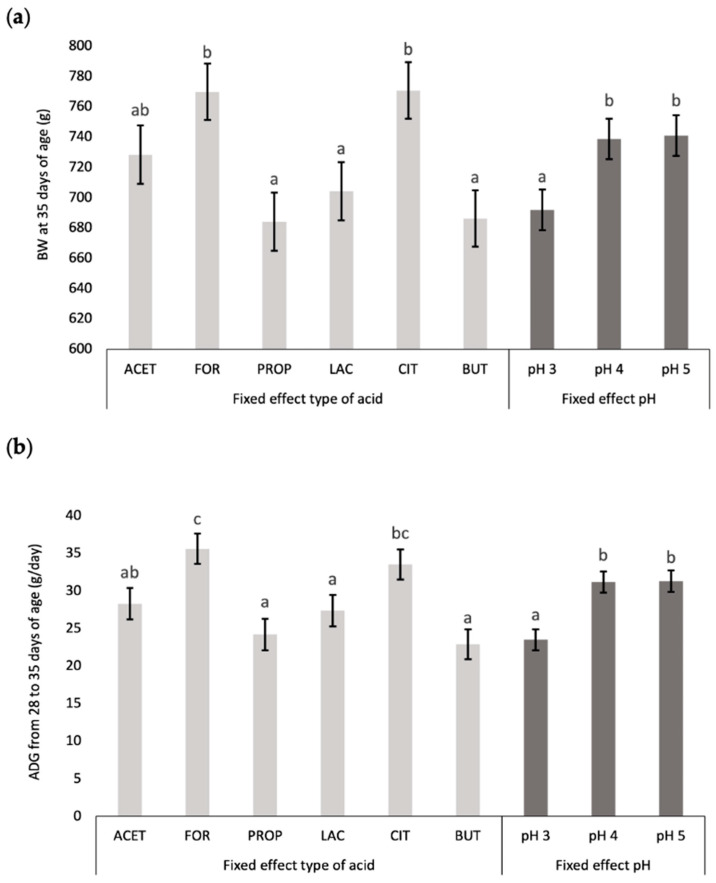
Comparison of the fixed effect (type acid and pH) on (**a**) body weight (BW) at 35 days of age (*n* = 18 animals per acid type, and *n* = 36 animals per pH level), and (**b**) average daily gain (ADG) from 28 to 35 days of age (*n* = 18 animals per acid type, and *n* = 36 animals per pH level). Acetic acid (ACET), formic acid (FOR), propionic acid (PROP), lactic acid (LAC), citric acid (CIT), and butyric acid (BUT). Results with different letters for fixed effect (type of acid and pH) differ significantly from each other (*p* < 0.05).

Considering the fixed effects described in Table 2, the type of acid and pH significantly influenced final BW at 35 days of age and global ADG (from 28 to 35 days of age). Figure 4 illustrates the effect of acid type (ACET, FOR, PROP, LAC, CIT, and BUT) and pH level (pH 3, 4, and 5) on these variables. With regards to acid type, our results indicate the use of FOR and CIT acids allowed to reach greater BW at 35 days of age and global ADG compared to PROP, LAC, and BUT (*p* < 0.05); showing ACET intermediate values. With regards to pH level, animals drinking acidified water at pH 4 and pH 5 showed higher BW at 35 days of age and global ADG compared to pH 3 (*p* < 0.05).

### 3.3. Effect on Digestive Physiological Measurements: Gastrointestinal pH and Gastric Pepsin

Table 3 shows the pH levels along the gastrointestinal tract and gastric pepsin activity for all experimental treatments compared to the CON group. Initial pH at weaning was 2.97 for the fundus, 1.40 for the antrum, 7.20 for the duodenum, 7.45 for the jejunum, 7.69 for the ileum, and 5.76 for the cecum. After weaning, average pH values, independent from the treatment, were 3.72 and 3.90 for the fundus, 1.51 and 1.54 for the antrum, 7.28 and 7.09 for the duodenum, 7.55 and 7.42 for the jejunum, 7.70 and 7.55 for the ileum, and 5.69 and 5.62 for the cecum, for 31 to 35 days of age, respectively. In comparison to CON, OAs showed a limited capacity to reduce gastric pH and pH in the proximal segments of the small intestine. The addition of some OAs even resulted in a slight increase in pH values. Therefore, a significantly higher pH than the CON group (*p* < 0.05) was obtained for FOR 3, PROP 5, LAC 4 and CIT 3 treatments in the fundus, and for CIT 4 and CIT 5 in the antrum. At the intestinal level, pH in the duodenum was significantly lower in CIT 3 compared to CON (−0.22, *p* = 0.0216). The pH in the jejunum for PROP 3 and in the caecum for BUT 3, were significantly higher than CON. There were no statistically significant differences among the rest of experimental treatments with CON group in gastrointestinal pH values.

The addition of OA did not increase pepsin enzyme activity compared to the CON group (Table 3). In most of cases, pepsin activity was not significantly different to those observed in CON group (*p* > 0.05). After weaning, average gastric pepsin activity was 108.9 and 117.4 pepsin units at 31 and 35 days of age, respectively. However, treatments ACET 3, FOR 3, PROP 3, PROP 5, LAC 3, CIT 5, and BUT 5 significantly decreased pepsin enzyme activity compared to the CON group (−101,9, −103.8, −59.9, −118.5, −100,9, −108.5, and −69.7 pepsin units, respectively; *p* < 0.05). 

**Figure 5:**
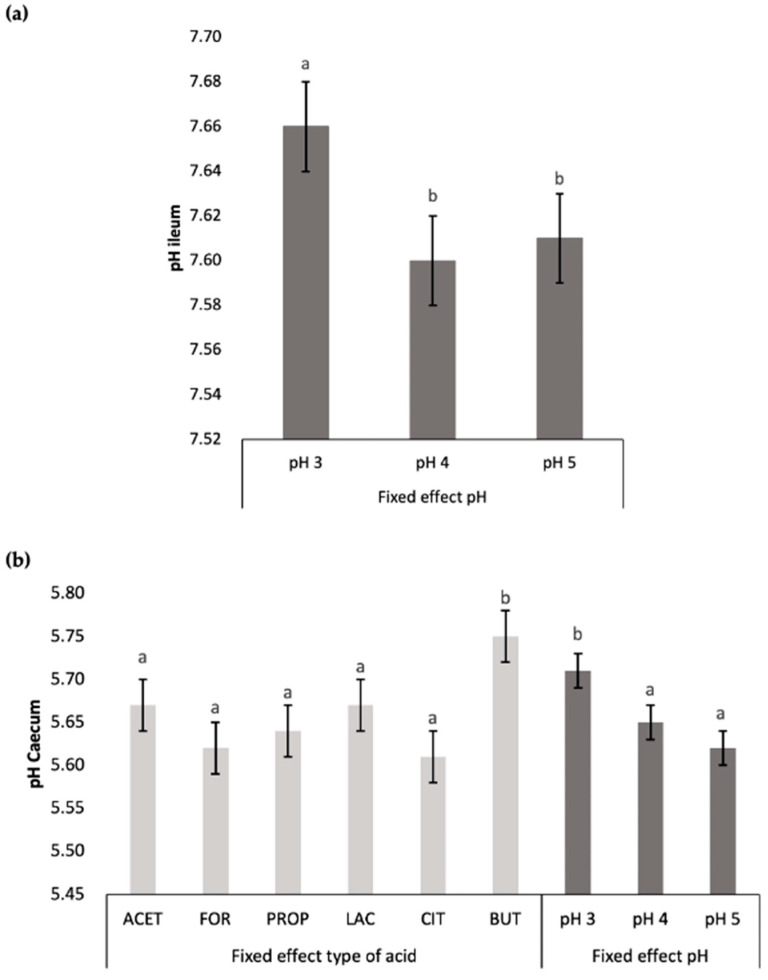

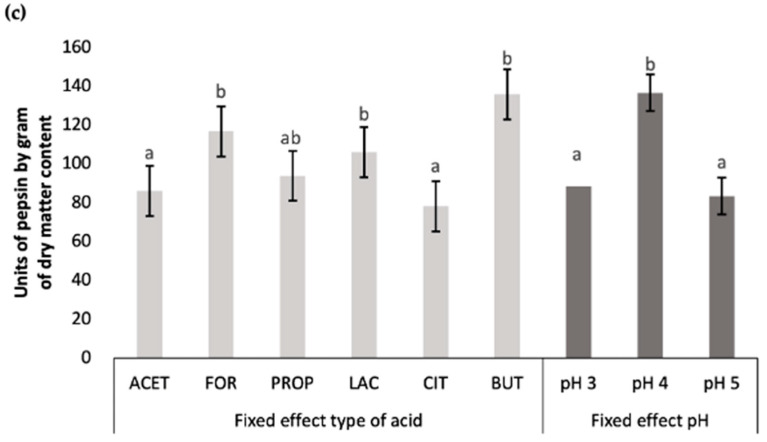
Comparison of the fixed effect (type acid and pH) on (**a**) ileum pH, (**b**) caecum pH, and (**c**) gastric pepsin activity. Acetic acid (ACET), formic acid (FOR), propionic acid (PROP), lactic acid (LAC), citric acid (CIT), and butyric acid (BUT); *n* = 36 animals per acid type and *n* = 72 animals per pH level. Results with different letters for fixed effect (type of acid and pH) differ significantly from each other (*p* < 0.05).

Considering the fixed effects described in Table 3, the type of acid and water pH significantly affected ileum pH, caecum pH and pepsin activity. Figure 5 illustrates the effect of acid type (ACET, FOR, PROP, LAC, CIT, and BUT) and water pH level (pH 3, 4, and 5) on some of these variables. Regarding pH values, animals drinking acidified water at pH 4 and pH 5 showed lower (*p* < 0.05) ileum and caecum pH values compared to pH 3 (Figure 5a,b). Additionally, animals drinking acidified water at pH 4 showed the highest (*p* < 0.05) pepsin activity compared to pH 3 and pH 5 (Figure 5c). Regarding acids, our results indicate the use of BUT significantly increased caecum pH compared to the rest of acids (Figure 5b). Pepsin activity was higher in FOR, LAC, and BUT compared to ACET and CIT, while PROP showed intermediate values (Figure 5c).

## 4. Discussion

Our study serves as a screening design to provide insights into the most suitable OAs and those that should be eliminated due to negative effects at an early phase in growing rabbits. Therefore, as a screening test, the main goal is to gather initial animal’s responses that can be further validated in subsequent research on a larger scale with higher number of animals. This initial exploration is crucial for refining research, in accordance with the principles of the 3Rs (Replacement, Reduction, and Refinement) in animal science. Our approach, which includes an extensive number of combinations, despite the low number of replicates for some variables (particularly ADWI and ADFI), aims to gather qualitative information and interactions that may influence the selection of the most appropriate OA to be further validated. For this reason, we administered OAs via drinking water, due to its ease of handling, especially considering the high number of OA and dosages (pH) used.

In rabbit farming, weaning introduces a number of stressors related to changes in feed, social interactions, and exposure to pathogens. This stress can lead to different behavioural and physiological changes. Such alterations can result in immunosuppression [29], thereby increasing the likelihood of developing digestive disorders. It is evident that variations in diet can result in notable physiological alterations at the digestive level [11]. For instance, intake of either milk or solid feed can highly influence gastric pH. During the first 29 days of age, stomach pH can be high (>4.5), mainly due to limited hydrochloric acid secretion, the basic pH of milk, and its buffering capacity [30]. Subsequently, the pH declines (ranging from 4.6 to 1) as the gastric system matures, coinciding in turn with the increase in solid feed intake [11,30]. Although the acid barrier of the stomach during lactation is not particularly robust, colonization of the digestive tract by microorganisms is relatively poor [11]. This is due to the richness in the medium-chain fatty acids of rabbit milk, caprylic acid (C8) and capric acid (C10), which possess antibacterial activity [31]. However, the antimicrobial protection provided by these medium-chain fatty acids is reduced after weaning, while the gastric acid barrier is still under development. Our results show an initial pH in the stomach varied between 1.40 and 2.97. These values are unusually low according to the literature [11]. However, the addition of OAs would reinforce and help maintain this pH in addition to helping inhibit microbial growth in the stomach [18,19].

Using OAs can help strengthen the gastric barrier, enhance nutrient digestion in growing rabbits, and mitigate the colonization of pathogenic microorganisms in the digestive system. However, the results of their use in rabbit farming are scarce and sometimes contradictory [18]. For instance, Michelan et al. [32] included fumaric acid (1.5 g/kg feed) in growing rabbits’ diet and observed a slight numerical increase in BW, ADG, ADFI, and feed conversion rate (FCR) compared to the control; but differences were not statistically significant. Scapinello et al. [33] did not observe significant differences in performance or mortality when including four dosages (0.5, 1, 1.5, 2 g/kg feed) of acetic or fumaric acid. Other studies, however, have observed significant positive effects of water acidification on rabbit performance. Zhu et al. [34] tested a commercial OA mixture (containing a mix of formic acid, acetic acid and ammonium formate) at three different dosages in drinking water (0.55 g/kg—pH 5, 0.85 g/kg—pH 4.3 and, 3.3 g/kg—pH 3.6) in growing rabbits during a whole rearing cycle (until 70 days of age). In this study, water treatment at pH 4.3 significantly improved ADG, FCR, and final BW compared to the control. In contrast, Cesari et al. [35], did not find improvements in final BW, feed intake, and health status when using a mixture of formic and lactic acid (5 g/kg) via feed. However, rabbit performance improved from 55 to 84 days of age. More recently, Chowdhury et al. [36] also found no improvement in performance with the addition of citric acid, but improved crude protein digestibility probably by improving enzyme activity. The addition of citric acid in feed did improve feed utilisation by improving ADG and FCR. Abdl Razek Mohmed et al. [37] and Chowdhury et al. [36] also reported an improvement in FCR when humic acid was added to the feed (at 0.35 g/kg, 0.70 g/kg and 1.05 g/kg). The aforementioned authors attributed the observed improvements in final BW, ADG, and FCR to enhanced feed digestibility or elevated pepsin activity [34,35,36,37]. Moreover, OA were administered for a longer duration than in our study. The duration of our study (7 days), difficulted a precise evaluation of productive performance due to the limited duration of the trial.

Our data demonstrates that reinforcing the acid barrier of the rabbit’s stomach with OAs is a complex process. The required dosage will depend on the physicochemical characteristics of the OA used. Nevertheless, this dosage may have an undesirable effect on the animal. In our study, despite the limited number of replicates used, water intake was influenced by the type of acid and dosage level. The results suggest that including OAs in drinking water at pH 3 may reduce water intake and affect feed consumption, thereby limiting the growth of the rabbits. It particularly occurred with the treatments where acetic, propionic or butyric acid were added to the drinking water at a high dosage to reach pH 3 (ACET 3, PROP 3, and BUT 3). For these treatments, we observed a 2.1 to 3.4-fold decrease in ADWI compared to CON. This reduction could compromise the overall health and growth performance of the animals, highlighting the importance of closely monitoring these parameters in the current screening phase instead of conducting large-scale experiments in the first run. 

This may be attributed to the organoleptic characteristics of these compounds, characterised by a strong taste and odour [38]. Zhu et al. [34] also observed a decrease in feed and water intake for low pH (3.6) in the drinking water, although no significant differences were found. These authors reported water intake of 172.6 mL/day for that treatment. In contrast, our results showed a lower water intake of 100 mL/day for ACET 3, PROP 3, and BUT3 groups (considering that the consumption data were derived from *n* = 2 cages per treatment). These intakes were even lower than those observed by Taha et al. [39] of 158.6 to 210.7 mL/day in growing rabbits of 35 days of age subjected to water restriction to two or three hours per day. According to these results, these OA at pH 3 should be avoided. Water intake conditioned the feed intake in these three groups in the same way because feed and water consumption are highly and positively related [39]. 

With regards to the acidification potential of OA in animal’s gut, there is also a considerable divergence in the literature. Zhu et al. [34] succeeded in acidifying the gastric content of rabbits by acidifying the drinking water to pH 4.3 and 5. However, the same effect in gastric pH was not achieved when the pH of the water reached 3.6, which may have been due to a reduction in water consumption. Cesari et al. [35] also observed a decrease in the caecal pH when a mixture of formic and lactic acid was added to the feed, compared to the control group. In contrast, Romero et al. [40] and Romero et al. [41] were unable to reduce the caecal pH compared to the control group by adding a mixture of formic and citric acid to the feed (at 0.4 and 0.2 g/kg, respectively) or by adding caprylic and caproic acid to the feed.

Weak OAs are compounds with a pKa between 3.7 and 4.9. Consequently, their acidic effect is diminished when they are present in media with a pH below 3.7. This is due to the fact that at pH values below their pKa, these compounds maintain a higher proportion of undissociated molecules. This could explain the limited stomach acidification capacity of the tested OAs, as reported by Romero et al. [40] and Romero et al. [41]. In the stomach, none of the acids at any of the three pH levels used in our study significantly lowered the antrum and fundus pH compared to the CON group. This could also be partially attributed to the considerable variation in pH observed across different locations within the gastric cavity, mainly in the fundus (showing the highest standard errors). Gidenne and Fortun-Lamothe [11] described fundus pH as more neutral, as caecotrophy is stored, whereas antrum pH is commonly very acidic (pH < 3). In our study, although pH measurements were conducted at 20:00 p.m., in order to minimise the influence of caecotrophy, it was observed that some animals exhibited the presence of caecotrophs in the fundus. Moreover, while sampling time was selected to minimise caecotrophy and respecting rabbit’s nychthemeral feed intake pattern, feed’s buffering capacity may also have influenced the result. Buffer capacity of our experimental diet was 176 µmol H^+^/unit per g (following the methodology described in Mennah-Govela et al. [42]). 

After passing through the stomach, OAs reach the small intestine, where their acidifying potential increases due to the higher pH, often exceeding their pKa. Once in the intestinal lumen (with pH values of approximately 7.5), the OAs will dissociate, releasing protons and reducing the pH of the small intestine. Our results indicate a slight decrease in pH in the duodenum with most of the OA treatments compared to CON, although this was not statistically significant. With regards to pH, the dose of pH 4 and 5 reduced the pH in the ileum compared to pH 3 (*p* < 0.05). Beyond this intestinal section, no clear differences were observed in the measured pH due to the use of OA. This can be explained because these compounds are largely absorbed in the duodenum as an energy source for enterocytes and other cellular processes [43]. On the other hand, duodenum also secretes bicarbonate, which neutralises the acidity of the bolus coming from the stomach and subsequently absorbed in the jejunum [44]. Consequently, the neutralisation of acidity and the absorption of OA in the intestine serve to restrict their capacity to act in the small intestine. The coating and/or protection of OAs can be used to avoid their rapid absorption, thus overcoming this limitation. These protections can be either lipidic or in combination with aromatic compounds [43].

Subsequently in the cecum, inclusion of ACET, FOR, PROP, LAC, and CIT resulted in a reduction (*p* < 0.05) in pH compared to BUT. Regarding pH, the doses of pH 4 and 5 reduced the pH in the cecum compared to pH 3 (*p* < 0.05). The lower consumption of BUT3 treatment could have caused a smaller amount of digestive content reaching the cecum. This could cause a decrease in bacterial fermentation of the undigested feed [44], which releases volatile fatty acids (VFAs), decreasing pH in the rest of the treatments. One of the most frequent VFAs produced by this fermentation in rabbits is acetic acid [44].

Finally, as regards digestive enzymes, a reduction in gastric pH leads to an increase in gastric proteolytic enzyme activity [34]. In our case, all treatments and even the initial values obtained for gastric pH were low. Therefore, high and equal enzyme activity between treatments is to be expected. However, enzymatic activity is measured not only by pH but also by the concentration of hydrochloric acid, and the amount of substrate [45]. Our results showed that pepsin activity was significantly higher (*p* < 0.05) at pH 4 compared to other dosages. Although the pepsin activity values for ACET and CIT were globally lower than in CON, at pH 4, the enzymatic activity was equivalent to that of CON. 

## 5. Conclusions

The objective of this study was to screen combinations of acids and pH values to identify optimal candidates, while eliminating those that may exhibit early adverse effects. To do this, our experimental design included a short-term assessment period. According to rabbit’s BW and weight gain (from 28 to 35 days of age), the OAs with the highest potential for use in post-weaning rabbits were formic acid, acetic acid, and citric acid. Combining the results of ileum and cecum pH, and gastric pepsin activity, optimal pH value was pH 4. The selected formic acid, acetic acid, and citric acid treatments at pH 4 did not exhibit any early adverse effects in young rabbits. Even so, it seems that the acidification capacity of OAs at the gastric level is limited. However, these compounds have other beneficial pathways of action. Further research is needed to determine the most appropriate route of administration (water or feed) for these compounds. Additional trials with the selected OAs, extended application periods, larger sample sizes, and more replicates per treatment are recommended to evaluate their effects throughout the entire growing period in rabbits.

## Figures and Tables

**Table 1:** Organic acids used during the experiment.

Organic Acid	Physicochemical Characteristics	Reference
Acetic acid	Liquid; purity of 99.5%; pH 2.5; pKa 4.74	W200603 Sigma Aldrich (St. Louis, MO, USA)
Formic acid	Liquid; purity of 95%; pH 2.2 pKa 3.70	F0507 Sigma Aldrich (St. Louis, MO, USA)
Propionic acid	Liquid; purity of 99%; pH 2.5; pKa 4.88	1810 Panreac (Barcelona, Spain)
Lactic acid	Liquid; purity of 85%; pH 2.8.; pKa 3.86	W261106 Sigma Aldrich (St. Louis, MO, USA)
Citric acid	Solid powder; purity of 99%; pH 1,7; pKa 3.13	C0759 Sigma Aldrich (St. Louis, MO, USA)
Butyric acid	Liquid; purity of 99%; pH 2.0; pKa 4.90	B103500 Sigma Aldrich (St. Louis, MO, USA)

**Table 2:** Effect of the type of acid and pH level on body weight (BW, in g) and average daily gain (ADG, in g/day) when compared to water (CON) in young rabbits from 28 to 35 days of age.

Treatment	BW 28 Day	BW 31 Day	BW 35 Day	ADG 28–31 Day	ADG 31–35 Day	ADG 28–35 Day
CON	530	610	774	26.7	36.9	33.8
ACET 3	532	549*	674*	5.8*	39.4	23.8*
ACET 4	537	623	730	28.5	30.8	28.9
ACET 5	531	632	780	33.5	34.2	32.0
FOR 3	530	619	799	29.4	39.4	36.1
FOR 4	530	616	763	28.9	37.9	35.6
FOR 5	530	620	748	29.9	36.5	35.1
PROP 3	537	516*	600*	−6.9*	26.3	9.8*
PROP 4	526	624	743	32.5	34.0	34.2
PROP 5	537	633	709	32.0	29.5	28.8
LAC 3	533	610	748	25.8	30.7	30.0
LAC 4	522	603	692	27.1	23.5	25.1
LAC 5	525	599	673	24.9	33.3	27.3
CIT 3	541	635	784	31.5	36.0	33.8
CIT 4	535	603	749	22.6	35.0	30.7
CIT 5	532	638	778	35.1	34.8	35.8
BUT 3	523	463*	546*	−20.1*	31.9	7.6*
BUT 4	533	614	756	26.9	37.7	32.4
BUT 5	542	605	757	21.1	35.2	28.8
SEM ^1^	16.5	20.3	32.5	3.4	4.2	3.4
*p*-value	1.000	<0.0001	<0.0001	<0.0001	0.4923	<0.0001
*p*-value Batch	0.0012	0.0098	0.0027	0.8759	0.0004	0.0186
*p*-value Acid	0.9880	0.0029	0.0108	<0.0001	0.1176	<0.0001
*p*-value pH	0.9641	<0.0001	0.0203	<0.0001	0.9391	0.0002

* Indicates statistically significant differences of the treatment with CON group; *n* = 12 animals per treatment from 28 to 31 days of age, and *n* = 6 animals per treatment from 31 to 35 days of age and globally; *n* = 36 animals per acid type from 28 to 31 days of age, and *n* = 18 animals per acid type from 31 to 35 days of age and globally; *n* = 72 animals per pH level from 28 to 31 days of age, and *n* = 36 animals per pH level from 31 to 35 days of age and globally. The experimental treatments comprised: acetic acid (ACET 3, ACET 4, ACET 5), formic acid (FOR 3, FOR 4, FOR 5), propionic acid (PROP 3, PROP 4, PROP 5), lactic acid (LAC 3, LAC 4, LAC 5), citric acid (CIT 3, CIT 4, CIT 5), butyric acid (BUT 3, BUT 4, BUT 5), and control (CON). ^1^ SEM: standard error of the mean.

**Table 3:** Effect of the type of acid and pH level in the water and animals’ day of age on pH along the gastrointestinal tract and pepsin activity in the stomach when compared to water (CON) of young rabbits.

	Stomach		Intestine				
Treatment	Fundus	Antrum	Duodenum	Jejunum	Ileum	Caecum	Pepsin Activity ^2^
CON	3.39	1.47	7.22	7.44	7.62	5.64	164.80
ACET 3	3.85	1.50	7.26	7.53	7.70	5.70	62.87*
ACET 4	4.18	1.55	7.16	7.40	7.52	5.71	139.70
ACET 5	3.73	1.51	7.14	7.43	7.55	5.61	55.66*
FOR 3	4.29*	1.60	7.13	7.46	7.61	5.61	61.00*
FOR 4	4.08	1.58	7.15	7.54	7.63	5.62	135.5
FOR 5	3.15	1.44	7.26	7.44	7.62	5.62	152.6
PROP 3	3.20	1.46	7.29	7.56*	7.73	5.72	104.9*
PROP 4	3.99	1.52	7.01	7.50	7.45	5.61	127.2
PROP 5	4.49*	1.59	7.18	7.46	7.65	5.58	46.26*
LAC 3	4.08	1.61	7.15	7.51	7.62	5.67	63.91*
LAC 4	4.75*	1.56	7.15	7.46	7.57	5.65	161.4
LAC 5	3.60	1.51	7.24	7.48	7.62	5.69	92.86*
CIT 3	4.48*	1.54	7.00*	7.52	7.60	5.65	68.06*
CIT 4	3.80	1.64*	7.29	7.52	7.66	5.62	120.50
CIT 5	3.87	1.63*	7.08	7.48	7.56	5.55	56.26*
BUT 3	2.87	1.44	7.45*	7.50	7.69	5.89*	166.60
BUT 4	3.71	1.53	7.20	7.53	7.57	5.66	142.10
BUT 5	4.19	1.51	7.05	7.50	7.65	5.70	95.08*
SEM ^1^	0.32	0.17	0.08	0.04	0.04	0.05	20.84
*p*-value	0.0280	0.1290	0.0389	0.2544	0.0732	0.0009	<0.0001
*p*-value day	0.7211	0.1950	<0.0001	<0.0001	<0.0001	0.0001	0.5575
*p*-value batch	0.0425	0.6272	0.3801	0.1391	0.4694	0.0317	<0.0001
*p*-value Acid	0.4342	0.1249	0.7001	0.5466	0.1512	0.0023	0.0198
*p*-value pH	0.2340	0.3529	0.4681	0.0803	0.0404	0.0106	<0.0001

* Indicates statistically significant differences compared to the CON group; *n* = 12 animals per treatment; *n* = 36 animals per acid type and *n* = 72 animals per pH level. The experimental treatments comprised: acetic acid (ACET 3, ACET 4, ACET 5), formic acid (FOR 3, FOR 4, FOR 5), propionic acid (PROP 3, PROP 4, PROP 5), lactic acid (LAC 3, LAC 4, LAC 5), citric acid (CIT 3, CIT 4, CIT 5), butyric acid (BUT 3, BUT 4, BUT 5), and control (CON). ^1^ SEM: standard error of the mean. ^2^ Pepsin activity: units of pepsin per gram of dry matter content.

## Data Availability

Data are contained within the article. The datasets of the current study are available from the corresponding author upon reasonable request.

## References

[1] Licois D., Wyers M., Coudert P. (2005). Epizootic Rabbit Enteropathy: Experimental transmission and clinical characterization. Vet. Res..

[2] Hu B., Fan Z.-Y., Wei H.-J., Song Y.-H., Qiu R.-L., Chen M.-M., Xu W.-Z., Xue J.-B., Wang F. (2018). Detection of mucoid enteropathy syndrome disease in rabbit farms in East China. Res. Vet. Sci..

[3] Marlier D., Dewrée R., Lassence C., Licois D., Mainil J., Coudert P., Meulemans L., Ducatelle R., Vindevogel H. (2006). Infectious agents associated with epizootic rabbit enteropathy: Isolation and attempts to reproduce the síndrome. Vet. J..

[4] Huybens N., Houeix J., Licois D., Mainil J., Marlier D. (2009). Inoculation and bacterial analyses of fractions obtained from the reference inoculum TEC4 which experimentally reproduces the epizootic rabbit enteropathy. World Rabbit Sci..

[5] Combes S., Michilland R.J., Monteils V., Cauquil L., Soulié V., Tran N.U., Gidenne T., Fortun-Lamothe L. (2011). Postnatal development of the rabbit caecal microbiota composition and activity. FEMS Microbiol. Ecol..

[6] Wegener H.C. (2006). Use of antimicrobial growth promoters in food animals: The risks outweigh the benefits. Antimicrobial Growth Promoters. Where Do We Go from Here?.

[7] Fukuda R.K. (2012). Antimicrobial Resistance Global Report on Surveillance.

[8] European Parliament and the Council of the European Union Regulation (EU) 2019/6 of the European Parliament and of the Council of 11 December 2018 on Veterinary Medicinal Products and Repealing Directive 2001/82/EC, Official Journal of the European Union; 2019. b. https://eur-lex.europa.eu/legal-content/EN/TXT/PDF/?uri=CELEX:32019R0006&from=EN.

[9] Partanen K.H., Mroz Z. (1999). Organic acids for performance enhancement in pig diets. Nutr. Res. Rev..

[10] Roselli M., Finamore A., Britti M.S., Bosi P., Oswald I., Mengheri E. (2005). Alternatives to in-feed antibiotics in pigs: Evaluation of probiotics, zinc or organic acids as protective agents for the intestinal mucosa. A comparison of in vitro and in vivo results. Anim. Res..

[11] Gidenne T., Fortun-Lamothe L. (2002). Feeding strategy for young rabbits around weaning: A review of digestive capacity and nutritional needs. Anim. Sci..

[12] Tung C.M., Pettigrew J.E. (2006). Critical review of acidifiers. Anim. Sci..

[13] Donowitz G.L., Page M.L., Mileur B.L. (1986). Alteration of normal gastric flora in critical care patients receiving antacid and cimetidine therapy. Infect. Control Hosp. Epidemiol..

[14] Dewrée R., Meulemans L., Lassence C., Desmecht D., Ducatelle R., Mast J., Vindevogel H., Marlier D. (2007). Experimentally induced epizootic rabbit enteropathy: Clinical, histopathologicaI, ultrastructural, bacteriological and haematological findings. World Rabbit Sci..

[15] Gutiérrez I., Espinosa A., García J., Carabaño R., De Blas C. (2003). Effect of protein source on digestion and growth performance of early-weaned rabbits. Anim. Res..

[16] Gómez-Conde M.S., García J., Chamorro S., Eiras P., Rebollar P.G., Pérez de Rozas A., Badiola I., De Blas J.C., Carabaño R. (2007). Neutral detergent-soluble dietary fiber improves gut barrier function in 25-day-old weaned rabbits. J. Anim. Sci..

[17] Carabaño R., Navarro I.B., Chamorro S., García J., Ruiz A.G. (2008). New trends in rabbit feeding: Influence of nutrition on intestinal health. Span. J. Agric. Res..

[18] Falcão-e-Cunha L., Castro-Solla L., Maertens L., Marounek M., Pinheiro V., Freire J., Mourao J.L. (2007). Alternatives to antibiotic growth promoters in rabbit feeding: A review. World Rabbit Sci..

[19] Diebold G., Eidelsburger U., Barug D., de Jong J., Kies A.K., Verstegen M.W.A. (2006). Acidification of diets as an alternative to antibiotic growth promoters. Antimicrobial Growth Promoters.

[20] Tsiloyiannis V.K., Kyriakis S.C., Vlemmas J., Sarris K. (2001). The effect of organic acids on the control of post-weaning oedema disease of piglets. Res. J. Vet. Sci..

[21] Busser E.V.D., Dewulf J., Zutter L.D., Haesebrouck F., Callens J., Meyns T., Maes W., Maes D. (2011). Effect of administration of organic acids in drinking water on fecal shedding of *E. coli*, performance parameters and health in nursery pigs. Vet. J..

[22] Khan S.H., Iqbal J. (2016). Recent advances in the role of organic acids in poultry nutrition. J. Appl. Anim. Res..

[23] Chowdhury R., Islam K.M.S., Khan M.J., Karim M.R., Haque M.N., Khatun M., and Pesti G.M. (2009). Effect of citric acid, avilamycin, and their combination on the performance, tibia ash and immune status of broilers. Poult. Sci..

[24] Sánchez J.P., Theilgaard P., Mínguez C., Baselga M. (2008). Constitution and evaluation of a long-lived productive rabbit line. J. Anim. Sci..

[25] de Blas J.C., Mateos G.G., de Blas C., Wiseman J. (2020). Feed Formulation. Nutrition of the Rabbit.

[26] Merino J., Carabaño R. (2003). Efecto de la cecotrofia sobre la composición química de la digesta y sobre la digestibilidad ileal. ITEA.

[27] Rick W., Fritsch W.P., Bergmeyer H.U. (1974). Chymotrypsin, trypsin and pepsin. Methods of Enzymatic Analysis.

[28] SAS Institute (2009). SAS/STAT® 9.2 User’s Guide.

[29] Broom D., Johnson K. (1993). Stress and Animal Welfare.

[30] Orengo J., Gidenne T. (2007). Feeding behaviour and caecotrophy in the young rabbit before weaning: An approach by analysing the digestive contents. Appl. Anim. Behav. Sci..

[31] Cole C.B., Scott K.J., Henschel M.J., Coates M.E., Ford J.E., Fuller R. (1982). Trace nutrient binding proteins in milk and the growth of bacteria in the gut of infant rabbits. Br. J. Nutr..

[32] Michelan A.C., Scapinello C., Natali M.R.M., Furlan A.C., Sakaguti E.S., Faria H.G., Santolin M.L.R., Hernandes A.B. (2002). Utilization of probiotic, organic acid and antibiotic in diets for growing rabbits: Essay of digestibility, evaluation of intestinal morphometry and performance. Rev. Bras. Zootec..

[33] Scapinello C., Faria H.G., Furlan A.C., Pedro M.R.S. (1998). Influence of different levels of fumaric acid or acetic acid on the growing rabbits performance. Rev. Bras. Zootec..

[34] Zhu K.H., Xu X.R., Sun D.F., Tang J.L., Zhang Y.K. (2014). Effects of drinking water acidification by organic acidifier on growth performance, digestive enzyme activity and caecal bacteria in growing rabbits. Anim. Feed Sci. Technol..

[35] Cesari V., Toschi I., Pisoni A.M., Grilli G., Cesari N. Effect of dietary acidification on growth performance and caecal characteristics in rabbits. Proceedings of the Ninth World Rabbit Congress.

[36] Chowdhury R., Rahman M.A., Al-Mamun M. (2022). Influence of dietary organic acid, probiotic and antioxidant on the growth performance and nutrient digestibility in growing rabbit. Bang. J. Anim. Sci..

[37] Abdl Razek Mohmed M.S., Elsebai A., Elghalid O.A., Abd El-Hady A.M. (2020). Productive performance, lipid profile and caecum microbial counts of growing rabbits treated with humic acid. J. Anim. Physiol. Anim. Nutr..

[38] Costa L.B., Luciano F.B., Miyada V.S., Gois F.D. (2013). Herbal extracts and organic acids as natural feed additives in pig diets. S. Afr. J. Anim. Sci..

[39] Taha A.E., Rashed R.R., Hassan S.S. (2014). Impact of water restriction on the productive and behavioral performance of two fattening rabbit breeds. Global Vet..

[40] Romero C., Rebollar P.G., Dal Bosco A., Castellini C., Cardinali R. (2011). Dietary effect of short-chain organic acids on growth performance, mortality and development of intestinal lymphoid tissues in young non-medicated rabbits. World Rabbit Sci..

[41] Romero C., Rebollar P.G., Moscati L., Dal Bosco A., Castellini C., Cardinali R. (2012). Effect of substitution of medium-chain organic acids for zinc bacitracin in a diet containing colistin on performance and development of intestinal lymphoid tissues in growing rabbits experimentally infected with *Escherichia coli* O103 and *Clostridium perfringens* toxinotype A. Anim. Feed Sci. Technol..

[42] Mennah-Govela Y.A., Singh R.P., Bornhorst G.M. (2019). Buffering capacity of protein-based model food systems in the context of gastric digestion. Food Funct..

[43] Tugnoli B., Giovagnoni G., Piva A., Grilli E. (2020). From acidifiers to intestinal health enhancers: How organic acids can improve growth efficiency of pigs. Animals.

[44] Johnson-Delaney C.A. (2006). Anatomy and physiology of the rabbit and rodent gastrointestinal system. Proc. Assoc. Avian. Vet..

[45] Prince N.C., Stevens L. (1999). Fundamentals of Enzymology: The Cell and Molecular Biology of Catalytic Proteins.

